# Generation of Functional Dopaminergic Neurons from Reprogramming Fibroblasts by Nonviral-based Mesoporous Silica Nanoparticles

**DOI:** 10.1038/s41598-017-18324-8

**Published:** 2018-01-08

**Authors:** Jen-Hsuan Chang, Ping-Hsing Tsai, Kai-Yi Wang, Yu-Ting Wei, Shih-Hwa Chiou, Chung-Yuan Mou

**Affiliations:** 1 Department of Chemistry, National Taiwan University, Taipei, 106 Taiwan; 2 Department of Medical Research, Taipei Veterans General Hospital, Taipei, 112 Taiwan; 3 Institute of Pharmacology, National Yang-Ming University, Taipei, 155 Taiwan; 4 Institute of Neuroscience, National Yang-Ming University, Taipei, 155 Taiwan

## Abstract

Direct-lineage conversion of the somatic cell by reprogramming, in which mature cells were fully converted into a variety of other cell types bypassing an intermediate pluripotent state, is a promising regenerative medicine approach. Due to the risk of tumorigenesis by viral methods, a non-viral carrier for the delivery of reprogramming factors is very desirable. This study utilized the mesoporous silica nanoparticles (MSNs) as a non-viral delivery system for transduction of the three key factors to achieve conversion of mouse fibroblasts (MFs) into functional dopaminergic neuron-like cells (denoted as fDA-neurons). At the same time, a neurogenesis inducer, ISX-9, was co-delivered with the MSNs to promote the direct conversion of neuron-like cells. Good transfection efficiency of plasmid@MSN allowed repeated dosing to maintain high exogenous gene expression analyzed by qPCR and the changes in neural function markers were monitored. To further validate the dopaminergic function and the electrophysiological properties of fDA-neurons, the results of ELISA assay showed the high levels of secreted-dopamine in the conditional medium and rich Na^+^/K^+^-channels were observed in the fDA-neurons on Day 22. The results demonstrated that MSN nanocarrier is effective in delivering the reprogramming factors for the conversion of functional dopaminergic neurons from adult somatic cells.

## Introduction

Recently, direct lineage reprogramming of somatic cells by introducing defined factors becomes more promising for clinical translation since this approach could avoid the teratoma risk from the differentiation of induced pluripotent stem cells^1–5^. In 2010, Vierbuchen *et al*. identified three factors, Ascl1, Brn2, and Myt1l, which are sufficient to efficiently direct reprogram Mouse fibroblasts (MFs) into functional neurons *in vitro* ^2^. Recent studies have shown the direct conversion of human fibroblasts to other specific neural cells, such as dopaminergic neurons, GABAergic neurons, and glutamatergic neurons^2,6–8^.

In addition, several groups have studied the direct generation of neurons using small molecules, which could modulate specific signaling cascades and induce gene expression in the cells^9–14^. Li *et al*. (2015) studied the direct generation of functional neurons from fibroblasts using a cocktail of small molecules^12^. They found that the neurogenesis inducer, ISX-9 (Isoxazole 9), play an important role to activate the neuron-specific genes^15^. ISX-9, a synthetic promoter of adult neurogenesis, can trigger neuronal differentiation of adult neural stem/precursor cells. Hence a combination of the small molecule ISX-9 and plasmid for the direct conversion formation of neuron cells would be a good approach.

However, for future therapeutic direct conversion of cells, a limitation that needs to be overcome is that the introduction of viruses into human subjects is generally undesirable. In general, viral vectors mediate efficient gene transfer but a long-term expression of genes risks tumorigenicity which is of serious concern due to the gene integration into chromosome during the viral infection^16,17^. This substantiates the need to develop non-viral means of delivering reprogramming factors. In such cases, often a small molecule factor could further help inducing the reprogramming of the cell. Thus, a non-viral nanocarrier for simultaneous delivery of small molecule drug and plasmid would be most desirable. At present, most of the studied non-viral nanocarriers are cationic polymers such as PEI. However, cytotoxicity and non-biodegradability might be harmful in long-term safety considerations for cationic polymer-based carriers^18–20^.

**Figure 1:**
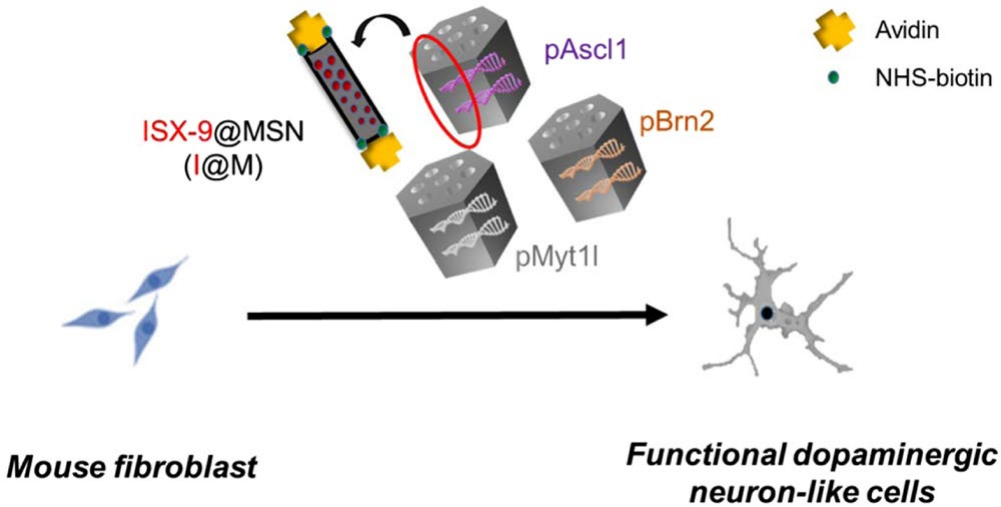
Ascl1 plasmid DNA (pAscl1), Brn2 plasmid DNA (pBrn2) and Myt1l plasmid DNA (pMyt1l) were complexed with the avidin-biotin-conjugated mesoporous silica nanoparticles with ISX-9 (pABM-I@M) by adsorption. The pABM-I@M was delivered into mouse fibroblast (MFs) to promote direct conversion of functional dopaminergic neuron-like cells.

Recently, mesoporous silica nanoparticles (MSNs) have been shown to be an excellent carrier for gene and drug delivery, taking advantage of its good biocompatibility, high surface area and easy surface functionalization^21–24^. For delivering plasmid, we recently applied mesoporous silica nanoparticles (MSNs) to co-deliver Nurr1 plasmid (pNurr1) and Rex1 siRNA (siRex1) to iPSCs (Induced pluripotent stem cells) to achieve dopaminergic neuron differentiation. However, such differentiation process from iPSC could have the limitations of long-term differentiation schedule and lower efficient rat of dopaminergic-positive cells. Thus, we would like to try a direct conversion approach using MSN nanocarrier as the direct-reprogramming technology. In this study, we demonstrate a non-viral delivery system based on MSN for plasmid and drug co-delivery for direct conversion of fibroblast cells to neurons. To design an effective controlled drug-delivery nanoparticle, we synthesized avidin capped-MSNs by avidin-biotin interaction for controlled release of the drug ISX-9^25^. In addition, the plasmid Ascl1, Brn2, and Myt1l are adsorbed on MSNs by electrostatic interaction (Fig. 1). The non-viral delivery system is tested for dual delivery of genes and hydrophobic drug into fibroblast to induce the direct conversion of MFs into functional dopaminergic neurons.

## Results and Discussion

### Characterizations of mesoporous silica nanoparticles

Mesoporous silica nanoparticles (MSNs) have been used extensively in nanomedicine, because of its good biocompatibility, high surface area (>1000 m^2^/g), tunable pore size and easiness surface functionalization. In this study, they were synthesized by our reported method^21^ and functionalized with the amine group (APTMS) to give MSN-NH_2_ to confer positive charge and then with biotin to give gated MSN-biotin. To develop a stimulation-response non-viral nanoparticle for loading and delivering ISX-9, the pores of MSN-biotin were capped by avidin with the strong biotin-avidin interaction to give MSN-avi. The three key differentiation factors, Ascl1, Brn2, and Myt1l, were adsorbed on the avidin-capped MSNs (abbreviated as MSN-avi) separately which was then delivered into the MFs at the same time. (Fig. 1)

**Figure 2:**
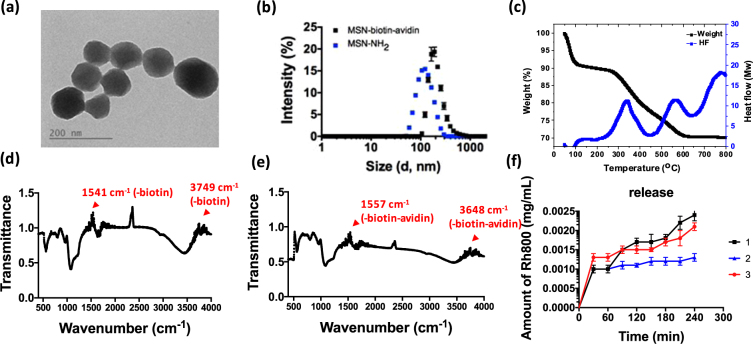
(**a**) The TEM image of MSN-avi, (**b**) the average dynamic light scattering (DLS) size of MSN-avi, (**c**) thermogravimetric analysis of MSN-avi, (**d**) Fourier transform infrared spectrum of MSN-biotin and (**e**) MSN-avi, (**f**) the release profile of Rh800@MSN-avi, 1: MSN-avi in which biotin was added after 1 hr, 2: MSN-avi without addition of biotin, and 3: MSN-biotin.

The average diameters of the nanoparticles, 3-aminopropyltrimethoxysilane (APTMS) modified MSNs (abbreviated as MSN-NH_2_) and MSN-avi, are 80~120 nm with the hexagonally arranged pore structure (TEM shown in Fig. 2a). The average sizes of MSN-NH_2_ and MSN-avi in aqueous solution are examined by dynamic light scattering (DLS) analysis. The average hydrodynamic diameters were determined by DLS (Fig. 2b), giving MSN-NH_2_ (122.8 nm, zeta = 30.4 mV) and MSN-avi (383.5 nm, zeta = −16.8 mV). They were well-dispersed in water. The hydrodynamical sizes and the zeta potential analysis of the nanoparticles are listed in Table S2. The weight percentage of the biomolecules (-NH_2_-biotin-NHS and the avidin protein) modified on the mesoporous silica nanoparticles were 8.02% and 11.45%, respectively as determined by thermogravimetric analysis (TGA) (Fig. 2c). In addition, the -NH_2_-biotin and –iotin-avidin modification on the MSNs was examined by the FTIR absorption band at 1541 and 1557 ${{cm}}^{-1}$ (Fig. 2d,e).

In testing the drug release, we used a small dye molecule rhodamine 800 (denoted as Rh800) as a probe to be released from MSN-avi during biotin-responsive drug release process (Fig. 2f). The Rh800 was loaded in the pores of MSN-NH2 and the biotin is added; after 1 hr they were capped with avidin. Figure 2f demonstrates that the dye Rh800 can be released upon addition of biotin competing for the avidin caps. From the release profiles, one can see that after an initial quick release (probably from the externally adsorbed Rh 800), there is little release of the Rh 800 afterwards. With uncapped MSN-biotin (no avidin), there is further release after 30 min to double the amount of total release. With the capped MSN-avi, there is a sudden release after triggering with added biotin (competing for avidin) and the total release is even higher than the uncapped MSN-biotin. The intracellular drug release could be triggered by the endogenous biotin molecule, for inducing the dissociation of avidin from MSN-avi^25^. The dissociation constant (*K*_*d*_) of avidin toward biotin molecule (~1 × 10^−15^ M) was lower than that of avidin and biotin-modified MSN (~3.5 × 10^−11^ M), which indicates that the endogenous biotin molecule could compete as an uncapping reagent^26^.

With the capping capability of MSN-avi checked, the neurogenesis drug ISX-9 was encapsulated in the MSN-avi, and the loading capacity of ISX-9 was determined to be 93.7 µmol/g MSN-avi, as analyzed by UV-vis spectrum (Table S3). We then used MSN-avi as a non-viral carrier for co-delivery of the three plasmid DNA, Ascl1, Brn2, and Myt1l, to achieve direct conversion of MFs into functional dopaminergic neuron-like cells. Herein, the pABM-I@M-induced functional dopaminergic neuron-like cell was denoted as iM-Neurons.

### Mesoporous silica nanoparticles uptaken by MFs

We first study the interaction between nanoparticles and MFs by examining the cellular uptake and the biocompatibility of nanoparticles. The MFs were incubated with 256 $\mu$g of the fluorescein isothiocyanate-labeled MSN-avi (FMSN-avi) for 1, 2, and 4 hrs. Flow cytometry data showed that the cellular uptake efficiency was 95.2% after 1 hr incubation (see Supplementary Fig. S2). The result indicated that FMSN-avi entered MF cells efficiently. Confocal microscopy analysis also showed that FMSN-avi was uptaken by MFs (see Supplementary Fig. S3) copiously. In Fig. S3, we also observed that some of the FMSN-avi (green channel) and the early endosome (red channel) could be co-localized in the merged image and some of the FMSN-avi have already escaped from the endosome and resided in the cytoplasm at 1hr (green dots in the merge channel). The data indicated that most of the FMSN-avi enter the cells through endocytosis mechanism.

### Mesoporous silica nanoparticles are nontoxic to iM-Neurons

To evaluate the *in vitro* cytotoxicity, we compared cell proliferation after loadings of the nanoparticles pABM-I@M. For comparison, we also examine the same plasmid factors loaded with Lipofectamine 2000 (pABM-Lipo); the cell viability was determined by counting the cell number. The cells were incubated with 256 $\mu$g of the pABM-I@M and or 6 μL/mL Lipofectamine 2000 for 4 hrs and then washed with PBS twice. The same amount of plasmid and ISX-9 was used in Lipofectamine 2000 and the mesoporous silica nanoparticle. The results showed that there was no significant reduction in cell viability and long-term cellular proliferations with MSN as carriers (pABM-I@M). However, Lipofectamine 2000-mediated transfection of pABM-Lipo resulted however in 72~80% of cell death after 7 days. The results indicated that mesoporous silica is a biocompatible nanocarrier compared to Lipofectamine 2000 for long-term iM-Neurons formation (see Supplementary Fig. S4).

### mRNA expression levels of neuron-related factors

**Figure 3:**
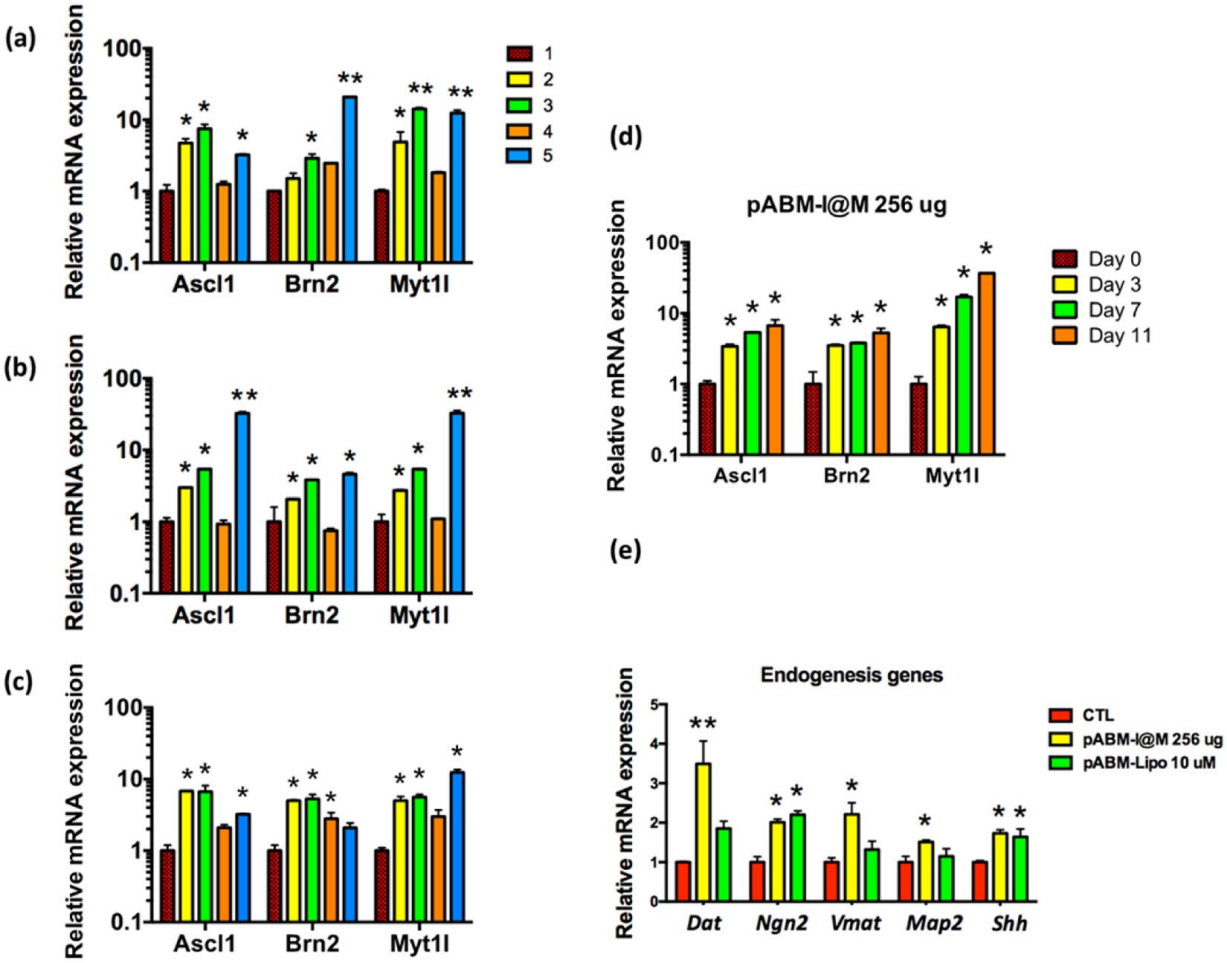
The endogenous messenger ribonucleic acid (mRNA) levels of Mouse fibroblasts (MFs) were determined after pABM-I@M and pABM-Lipo and controls. The (**a**)~(**c**) were indicated that the MFs were transfected with 1: the non-treatment condition, 2: 192 μg, 3: 256 μg of pABM factors complexed with I@M, 4: 256 μg of pABM factors complexed with MSN and 5: pABM factors complexed with Lipofectamine 2000. The mRNA levels were analyzed (**a**) after 3 days (one dose of pABM-I@M), (**b**) after 7 days (three doses pABM-I@M), and (**c**) after 11 days (five doses pABM-I@M), (**d**) the time-(0, 3, 7, 11 days) dependent mRNA levels under the treatment of pABM-I@M (256 μg), (**e**) endogenous mRNA levels of MFs were analyzed after 14 days and shown in after 14 days (five doses pABM-Lipo). Data are means ± standard deviation of three independent experiments. The asterisk (*) indicate statistical differences (p < 0.05) compared with control, and **indicate p < 0.001.

An equal amount of 2.0 $\mu$g pABM neural reprogramming factors in each of pABM-I@M (e.g. pA-I@M etc.) were delivered into MFs. Quantitative reverse transcriptase polymerase chain reaction (qRT-PCR) was used to examine the mRNA expression level of neuron-related genes enhanced by transfection of pABM-I@M. In Fig. 3, we show that the triple delivery of pAscl1, pBrn2, and pMyt1l was successfully transfected with I@M on Day 3, Day 7, and Day 11 and the dose-dependent and time-dependent effect of the three-corresponding mRNA were observed (Fig. 3d). As the time increased, the expression level of all the three-corresponding mRNAs also increased. On day 14 after the treatment of pABM-I@M, the qPCR analysis also shows the expression of neural function markers, Dat (Dopamine transporter, a symporter that moves dopamine across the cell membrane), Ngn2 (Neurogenin 2, played an important role in neurogenesis)^12^, Vmat (Vesicular monoamine transporter, acted to transport monoamine neurotransmitters), Map2 (Microtubule-associated protein 2, neuron-specific cytoskeletal proteins) and Shh (Sonic hedgehog, attracted commissural axons at the ventral midline of the developing spinal cord). Figure 3e shows the expression of Dat was 3.5-fold compared to control and the expression of Vmat was 2.2-fold compared to control after serial transfection of pABM-I@M on Day 14. However, the results for the Lipofectamine 2000 nanocarrier; the expression of Dat was only 1.9-fold compared to control, and the expression of Vmat was 1.3-fold compared to control after serial transfection of pABM-Lipo. The expressions of the other neuronal lineage markers, Ngn2, Map2, and Shh, were also shown that the serial transfection of pABM-I@M gave higher expressions of the mRNAs than that of Lipofectamine 2000 carrier. Thus, our MSNs carrier is more effective in promoting the gene transfection than using Lipofectamine 2000. Our data further supported that MSN-based nanocarrier system may provide a direct-conversion platform for adult somatic cells reprogramming into neurons.

### Significant cell morphology conversion

**Figure 4:**
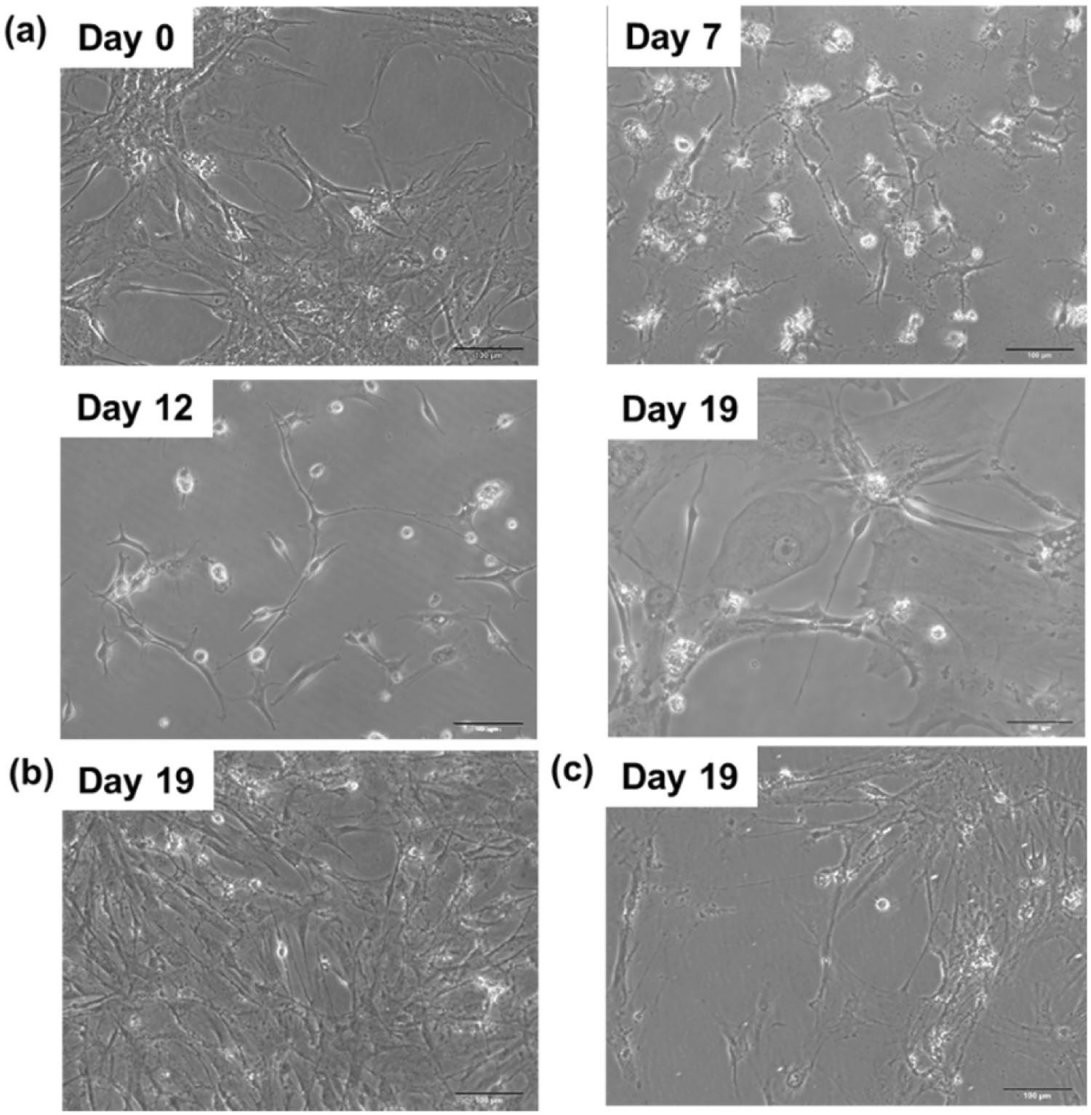
The images of bright field during direct conversion under the treatment of (**a**) pABM-I@M for 7 days, 12 days, and 19 days (256 µg/mL, pABM = 2 µg (total), transfection for 1 hr), (**b**) pABM-MSN-biotin for 19 days, and (**c**) pABM-Lipo + 10 µM ISX-9 for 19 days. (Lipofectamine 2000: 6 µL/mL, pABM = 2 µg (total), transfection for 4 hr). Scale bars are 100 μm.

Significant changes in cell morphology, such as the neural cell body, axon, and dendrite, are common signatures of neurons which were observed by microscope after iM-Neurons formation (Fig. 4a). As can be seen in Fig. 4, the more days pABM-I@M cells were treated the more the neuron-outgrowth morphology was. In comparison, for the pABM-MSN treated and the positive control condition (pABM-Lipo + 10 µM ISX−9), there are little neurite outgrowths. Accordingly, the neurogenesis drug, ISX-9, loaded in MSN-avi, promotes transfection efficiency of pABM and enhanced the maturation of iM-Neurons. One can observe substantial neurite outgrowths.

### The neural reprogramming factors promotes Tuj1+ and the expression of the neural lineage markers

**Figure 5:**
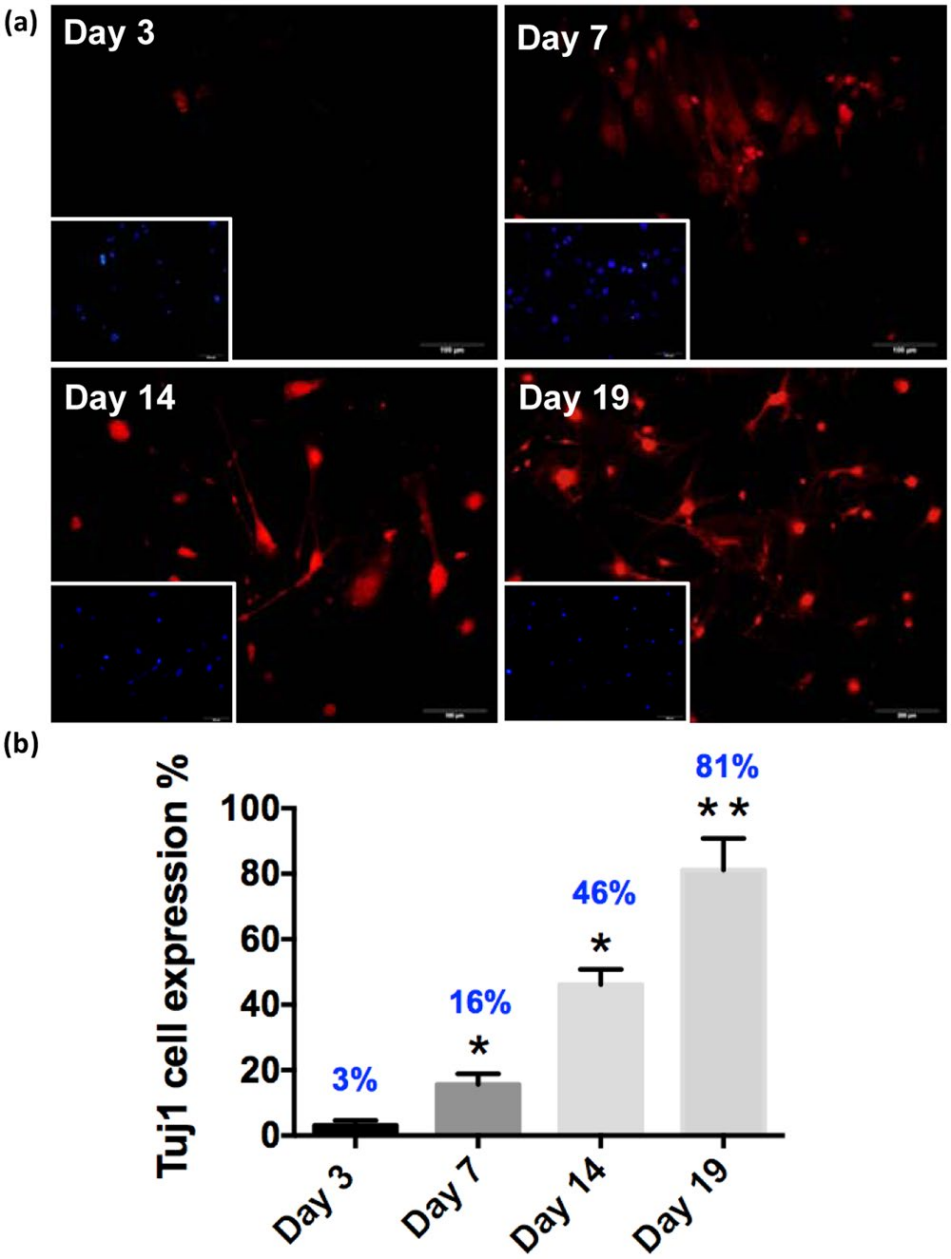
(**a**) Immunofluorescence staining was used to examine the expression of the Neuron-specific class III beta-tubulin (Tuj1, red) in pABM-I@M treated MFs after transfection for 3, 7, 14 and 19 days. (**b**) The image analysis-based quantification of the number of Tuj1-expressing cells was normalized to the number of nuclei which were counterstained with 4′,6-diamidino-2-phenylindole (DAPI, blue). Scale bars: 100 μm. Data are means $\pm$ standard deviation of three independent calculation of cell number ($\ge$200). The asterisk (*) indicate statistical differences (p < 0.05) compared with control, and **indicate p < 0.001.

To determine whether the neural progenitor marker of iM-Neurons was induced by pABM-I@M, immunofluorescence (IF) staining was used to determine the protein-level of neural progenitor marker. The IF staining of neuron-specific class III beta-tubulin (Tuj1), expressed in the neuronal cell bodies and processes, was observed and is shown in Fig. 5a for treatment with pABM-I@M on Day 3, 7, 14 and 19. The quantification of the number of Tuj1-expressing cells was normalized to the number of cells which were counter-stained with 4′,6-diamidino-2-phenylindole (DAPI, blue) as shown in Fig. 5b. The data showed that the increase of Tuj1-expressing cells was dominating 19 days after iM-Neurons formation; 81% of MFs were directly converted into neural progenitor cells.

**Figure 6:**
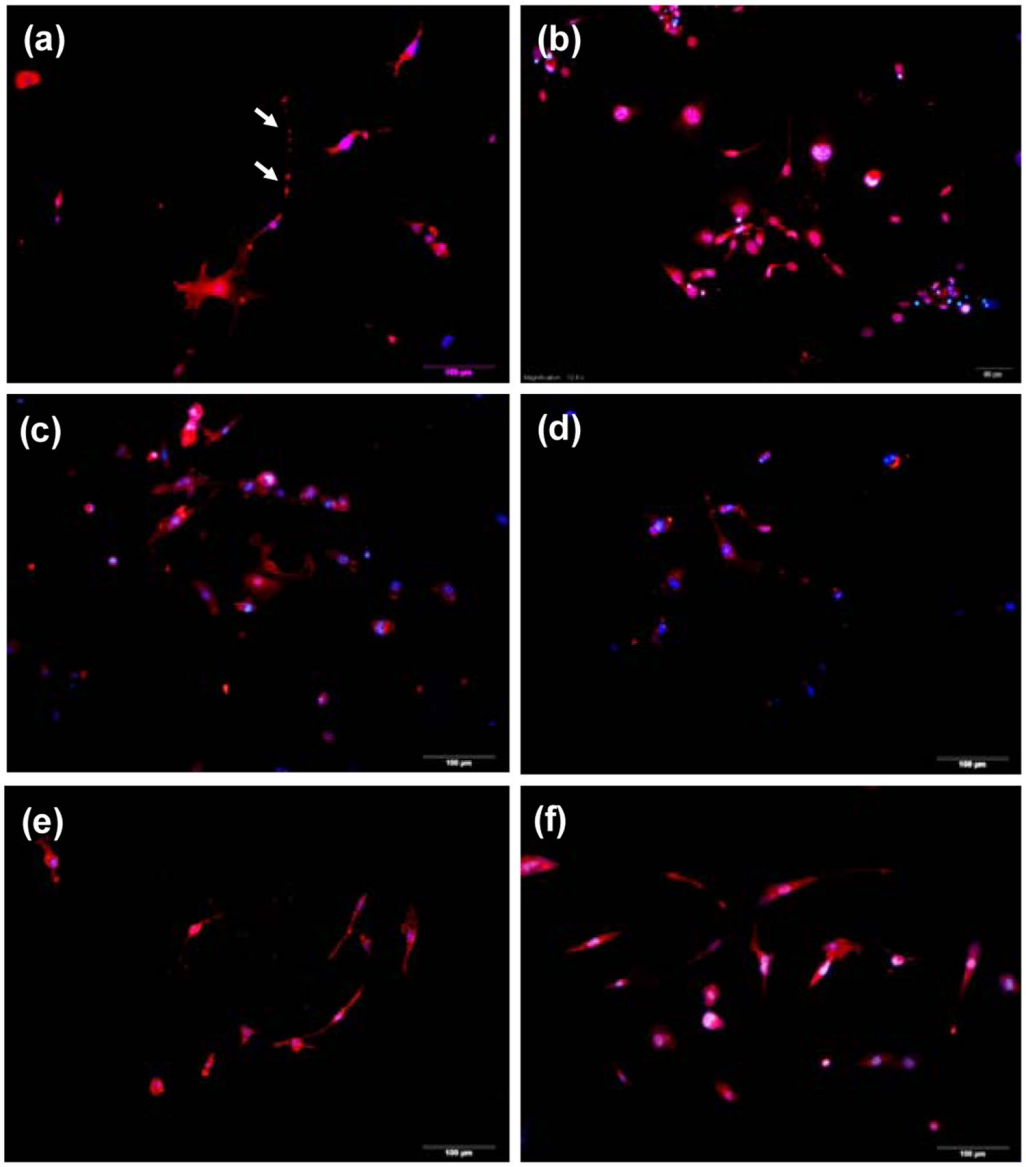
Immunofluorescence staining for the expression of the (**a**) synapsin I (red), (**b**) vesicular glutamate transporter 2 (VGlut2, red), (**c**) γ-Aminobutyric acid (GABA, red, (**d**) Tau protein (red), (**e**) tyrosine hydroxylase (Th, red), and (**f**) neuronal nuclei (NeuN, red) under the treatment of 256 µg/mL pABM-I@M for 19 days. The MFs were transfected for 1 hr) and the nuclei were counterstained with 4′,6-diamidino-2-phenylindole (DAPI, blue). Scale bars are 100 μm.

In order to examine the maturation of iM-Neurons, other neural lineage markers were also determined (Fig. 6). IF staining of Tau (proteins that stabilize microtubules), the protein marker expressed in post-mitotic stage of neuron: NeuN, dopaminergic neuron marker: Th (Tyrosine hydroxylase, a dopamine-related enzyme), GABAergic neuron marker: GABA, Glutamergic neuron marker: VGluT2, and the proteins of synaptic vesicles: synapsin I, were shown in Fig. 6. The synapsin I-positive puncta adjacent to dendrites of iM-Neurons was marked with white arrows. The qPCR data and IF staining analysis showed that the MFs was direct converted into various neuron-like cells by pABM-I@M. The MSNs are shown to be a good non-viral transfection reagent for multiple plasmid delivery.

### Enhanced expression of dopamine transporter and maturation of functional neurons

**Figure 7:**
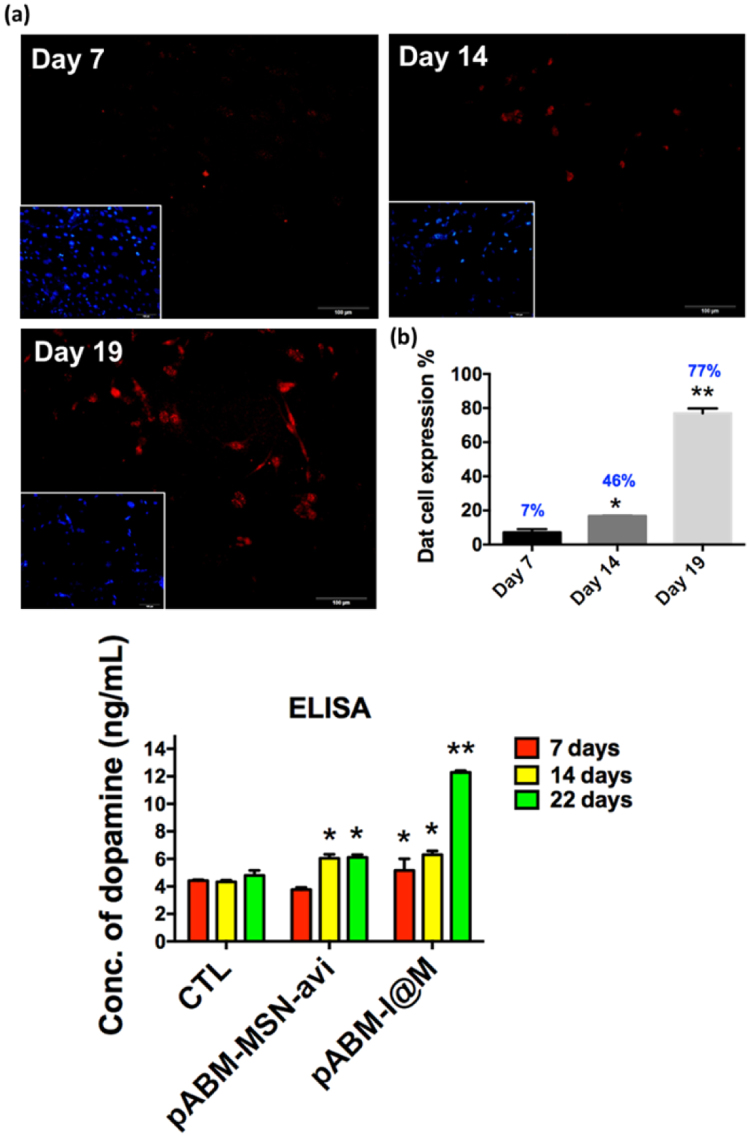
(**a**) Immunofluorescence (IF) staining was used to examine the expression of the Dopamine transporter (Dat, red) in pABM-I@M treated MFs after transfection 7, 14 and 19 days. (**b**) The image analysis-based quantification of the number of Dat-expressing cells was normalized to the number of nuclei which were counterstained with 4′,6-diamidino-2-phenylindole (DAPI, blue). (**c**) The quantification of dopamine analyzed by ELISA. Data are means ± standard deviation of three independent calculation of cell number ($\ge$200). The asterisk (*) indicate statistical differences (p < 0.05) compared with control, and **indicate p < 0.001.

To further examine whether the iM-Neurons present the characteristics of dopaminergic neurons, we monitored that the dopamine release ability from the iM-Neurons, which was analyzed by dopamine enzyme-linked immunosorbent assay kit (ELISA, LDN, Nordhorn, BA E-5300). One observes the neurotransmitter dopamine was released from neurons after iM-Neurons formation. In Fig. 7a, one observed increasing IF staining of the dopaminergic neuron marker on 7th, 14th and 22th days. The normalized percentage of Dat-expressing cells was 77% after 19 days iM-Neurons formation (Fig. 7b).

The dopamine was determined by a cis-diol-specific affinity gel with which the dopamine was acylated to N-acyldopamine and converted into N-acyl-3-methoxytyramine enzymatically during the detection. To detect dopamine, the antigen bound to the surface of the microtitor plate was examined by the anti-rabbit IgG-peroxidase conjugate and used 3,3,5,5′-Tetramethylbenzidine (TMB) as a substrate. In Fig. 7c, the average amount of dopamine secretion released from dopaminergic neuron cells was determined to be 5.0 ng/mL after the treatment of pABM-I@M on Day 7, and there were further increases in dopamine release at 7.49 ng/mL of dopamine, from neurons after the treatment of pABM-I@M on Day 22. There was no obvious increase of dopamine released from iM-Neurons in the non-treatment and pABM-MSN-avi controls in the iM-Neurons. The results showed that the high levels of secreted-dopamine in the conditional medium, which released from the cultured Day 22 fDA-neurons transfected by pABM-I@M, suggesting that these iM-Neurons exhibiting the dopamine-releasing function *in vitro*.

**Figure 8:**
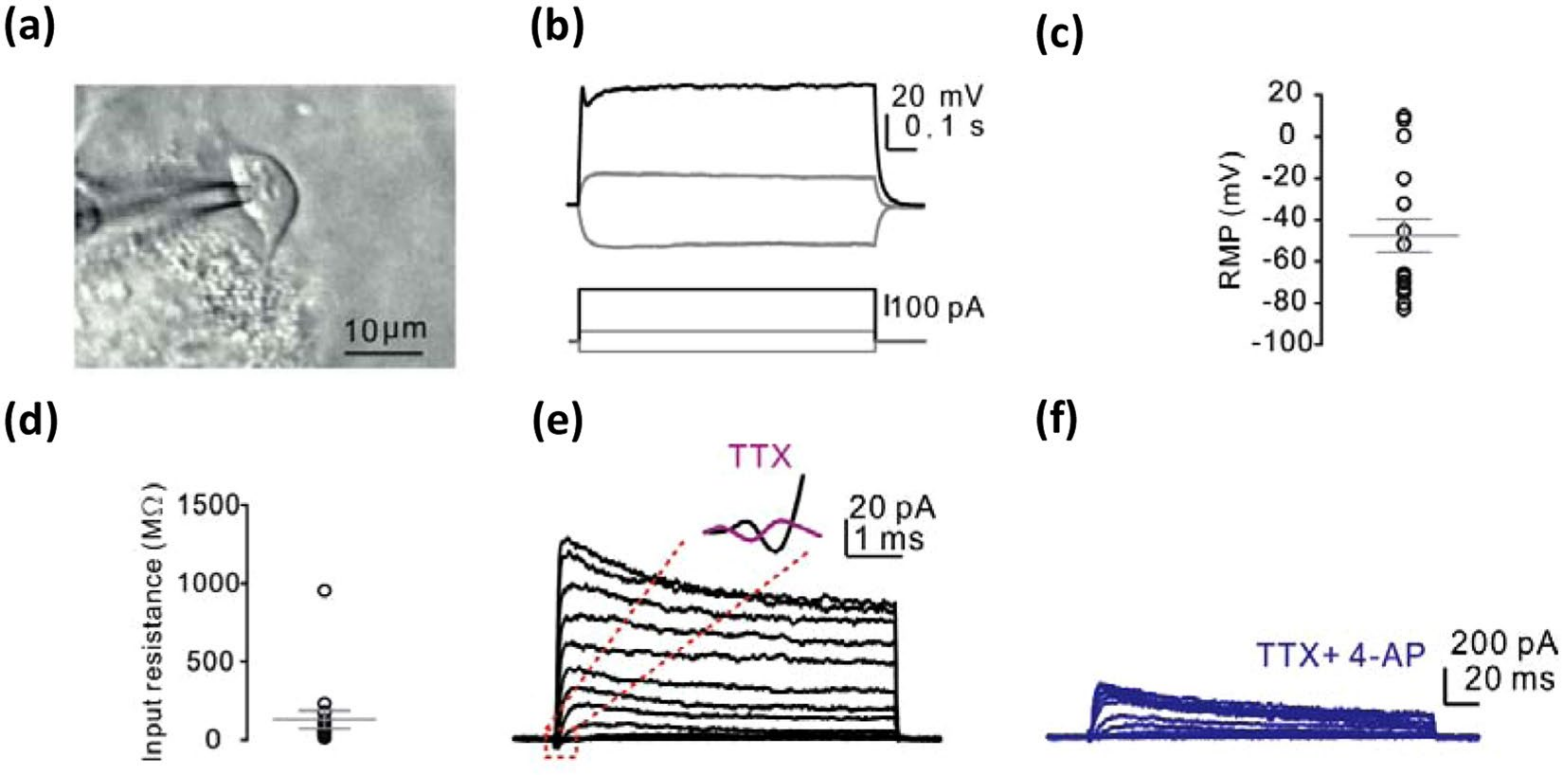
Electrophysiological properties of iM-Neurons: (**a**) IR-DIC image of patched MFs-neuron cell, (**b**) representative voltage responses during 1 s step-current injections, (**c**) summary of RMP of recorded cells (n = 16), (**d**) the input resistance of recorded cells (n = 16), (**e**) left, representative voltage-dependent Na^+^/K^+^ current during the voltage step; insect, the enlarged traces before (black) and after (purple) TTX application, (**f**) representative voltage-dependent Na^+^/K^+^ current in presence of TTX and 4-AP.

To examine whether the iM-Neurons perform signal transduction between cells, we perform a whole-cell patch-clamp assay to measure the electrophysiological properties within iM-Neurons. First, ovoid-shape iM-Neurons were applied to patch-clamp assay (Fig. 8a), and then we measure its putative process upon electric stimulation. The voltage response was observed in the recorded cells during the positive current injection (500 pA) (Fig. 8b). The average resting membrane potential (RMP) was −47.5 $\pm$ 8 mV (n = 16) and input resistance was 131 ± 56.85 MΩ (Fig. 8c,d). Besides, to investigate the properties of voltage-gated ion channels in the iM-Neurons, we gave the voltage step from 50 mV to −80 mV (Fig. 8e). Next, we added the voltage-gated Na^+^-channel blocker, tetrodotoxin (TTX) and the voltage-gated K^+^-channel blocker, 4-aminopyridine (4-AP) to confirm the signature of voltage-gated ion channels on iM-Neurons (Fig. 8f). These results indicated that there are voltage-gated Na^+^/K^+^-channels exist in the membrane of iM-Neurons, and MFs were directly converted into functional dopaminergic neuron-like cells induced by pABM-I@M^27^. In summary, these results indicated that iM-Neuron possess rich Na^+^/K^+^-channels and carried out electrophysiological transduction. We first demonstrated that MSN could be used to produce functional dopaminergic neuron-like cells from somatic cells.

## Conclusion

In summary, a non-viral delivery system of drug and plasmid for direct conversion of MFs into functional dopaminergic neuron-like cells was reported. Herein, we used mesoporous silica nanoparticles for the transduction of three factors, Ascl1, Brn2, Myt1l, into MFs together with the delivery of neurogenesis drug, ISX-9 to enhance the conversion of MFs-to-neuron. Biotin was functionalized on the of surface MSNs, and the avidin protein was used to encapsulate the ISX-9 into the MSNs (denoted as I@M). The drug-release was triggered by endogenous biotin molecule which could prolong and promote the efficacy of ISX-9. Immunofluorescence staining, ELISA assay, and the voltage-clamp recording analysis showed that the MFs were efficiently converting and reprogramming by pABM-I@M into functional dopaminergic neuron-like cells. The maturation of neurons was demonstrated by detection of dopamine production, rich Na^+^/K^+^-channels observed by the voltage-clamp study and a good population of neuron dendritic morphology. In addition, the low cytotoxicity and efficient delivery of the plasmids of I@M were demonstrated, and MFs-neuron could survive longer than cells treated by Lipofectamine 2000. We have shown that MSNs can act as a good biocompatible and non-viral carrier for transfection, and it could be applied to direct conversion of other specific lineages. Because of the targeting ability, nontoxicity and non-viral nature of MSNs, further studies on animals may be worthwhile. In the long-term, direct conversion approach using a safe non-viral nanocarrier may provide safe regenerative therapies for neurodegenerative disorders, including the Parkinsonian’s disease.

## Experimental section

### Synthesis of APTMS Conjugated Mesoporous Silica Nanoparticles, MSN-NH_2_

C_16_TABr (0.58 g, 1.64 × 10^−3^ mole) was dissolved in 200 mL aqueous ammonia solution (1.02 M) to which 5 mL of 0.21 M ethanol solution of TEOS (0.25 mL TEOS in 5 mL 99.5% ethanol) was added. After the solution was stirred at 40 °C for 1 hr, 0.88 M ethanol solution of TEOS (1.25 mL TEOS in 5 mL 99.5% ethanol) was added with vigorous stirring for 1 hr, and then it was standing at 40 °C for 24 hrs. The synthesized samples (MSNs) were then collected by centrifugation at 14000 rpm for 15 min, and it was washed with 99.5% ethanol for three times.

Next, the surfactant was extracted twice by ethanol solution of NH_4_NO_3_ (1 g NH_4_NO_3_ in 30 mL 99.5% ethanol) at 60 °C for 1 hr. Then, sample (MSN-ex) was collected by centrifugation at 14000 rpm for 15 min and washed with water twice and 99.5% ethanol three times. Afterwards, MSN-NH_2_ was obtained by modifying the MSN-ex surface with 3-Aminopropyl) trimethoxysilane (APTMS). Typically, 400 μL APTMS and 450 μL PEG-silane (MW 400) was mixed in 50 mL absolute ethanol containing 200 mg MSN-ex, and the mixture was refluxed at 80 °C for 12 hrs. Finally, MSN-NH_2_ was collected by centrifugation at 14000 rpm for 15 min and washed with 99.5% ethanol twice.

### Modification of MSN-NH_2_-biotin-avidin (abbreviated as MSN-avi)

4 mg of MSN-NH_2_, 400 λμL of sodium bicarbonate (0.1 M, pH = 8.4) and 2.67 mg of (+)-Biotin N-hydroxysuccinimide ester (in 26.67 μL DMSO) were mixed and stirred at 4 °C for 12 hrs. Then, MSN-NH_2_-biotin was collected by centrifugation and washed with 99.5% ethanol for three times. Next, MSN-NH_2_-biotin was dispersed in 1 mL phosphate-buffered saline (PBS) and 0.2 mg of avidin was added. Finally, the MSN-NH_2_-biotin-avidin was obtained by centrifugation and washed with PBS for three times.

### ISX-9/Rh800 loading

1 mg of MSN-NH_2_-biotin was mixed with 0.8 mM ISX-9 and stirred at 4 °C for 12 hrs. Then, 0.1 mg avidin was added and stirred for 1 hr. Then, samples were collected by centrifugation and washed with PBS for three times. The resulting materials are I@M. Similarly, a solution of 1 mg Rh800 in 1 mL PBS was used for loading of Rh800 in MSN-NH_2_ to obtain Rh800@M.

### Small molecule (Rh800) release

2 mg of Rh800@M was dispersed in 1 mL PBS buffer (pH = 7.4) at 37 °C. The release test was performed using a dialysis bag (Mw = 10 kDa). The released Rh800 could across the dialysis membrane but MSN-avi could not. The solution outside the dialysis membrane was collected every 30 minutes, and after 60 minutes, 0.6 mg of biotin was added into the solution. The released Rh800 from MSN-avi to the solution was examined by fluorescence emission at 710 nm.

### pABM-I@M Adsorption and Agarose Gel Electrophoresis

0.33 μg of pAscl1 was mixed with several weight ratios of I@M: 1/1, 1/2, 1/4, 1/8, 1/16, 1/32, 1/64, 1/128 in Dulbecco’s modified Eagle medium (DMEM) and incubated for 30 minutes, and the obtained conjugate is pA-I@M. Similar procedures were performed to load Brn2 and Myt1l to obtain pB-I@M and pM-I@M. And a final mixture of pA-I@M, pB-I@M and pM-I@M in the equal ratio is called pABM-I@M for the delivery of the three factors.

After mixing of the pA-I@M, pB-I@M and pM-I@M, pABM-I@M complexes were loaded in 1% agarose gel. After gel electrophoresis (at 110 V for 30 minutes), DNA bands were separated and visualized by ethidium bromide staining. The size standards, pAscl1, pBrn2, pMyt1l, and I@M were used as references.

### Cellular Cytotoxicity/ Cell proliferation

The Mouse fibroblasts (MFs) were treated with 6 μg/mL Lipofectamine 2000, 256 μg/mL pAscl1-pBrn2-pMyt1l-MSN (abbreviated as pABM-MSN), pAscl1-pBrn2-pMyt1l-MSN (pABM-I@M). After 4 hrs, the cells were washed with PBS and the cell cytotoxicity was examined by counting the cell number after the cells were trypsinized. In addition, the cell proliferation was analyzed at Day 7^th^.

### Flow cytometry analysis

The uptake of MSN-avi by MF was examined by flow cytometry as follows. MFs were seeded at 2 × 10^5^ cells per well in six-well plates and allowed to attach for 24 h. To determine the amount of FITC-conjugated MSN-avi (FMSN-avi) uptake by MFs, the cells were incubated with 256 μg/mL of FMSN-avi suspension in serum-free medium for 1, 2 and 4 h. Treated cells were then washed twice with phosphate-buffered saline (PBS), and then harvested by trypsinization. The green emitting fluorescein isothiocyanate dye in FMSN-avi serves as a marker to quantitatively determine their cellular uptake. In addition, trypan blue (ratio of volume, PBS:trypan blue = 6:1) was utilized to quench the FMSN that adsorbed on the membrane of MFs.

### FM4-64 staining of endosome after pABM-I@M delivery

The MFs were seed at a density of 1.5 × 10^5^ cells per cover slide well. After treatment of 256 $\mu$g of FMSN-avi for 1 hr, the cells were washed with PBS. Then, the cells were fixed in 1% paraformaldehyde and permeabilized with 0.1% NP-40. Finally, the nucleus was stained by 4′,6-diamidino-2-phenylindole (DAPI), and observed by confocal microscopy.

### MFs Transfection and generation of iM-Neurons

Before gene delivery, MFs were trypsinized into single cells and cultured in six-well plates. Next, delivery of 256 ${\rm{\mu }}$g pABM-MSN and pABM-I@M (weight ratio: pABM/MSN = 1/128) complexes into1 mL DMEM was carried out. Lipofectamine 2000 in DMEM (Invitrogen) was used as a positive control. Before delivery, 0.67 ${\rm{\mu }}$g of plasmid (pAscl or pBrn2 or pMyt1l) was mixed with 85.33 μg nanoparticle (MSN or I@M) in DMEM for 3 hrs, and the plasmid-nanoparticle complexes were formed. Finally, the pAscl1-MSN (or pAscl1-I@M), pBrn2-MSN (or pBrn2-I@M) and pMyt1l-MSN (or pMyt1l-I@M) were mixed together. The solution was added to six-well plates and the incubation time was 4 hrs. After washing with PBS, MFs were cultured in DMEM supplemented with 10% FBS, 1% of penicillin-streptomycin at 37 °C. Then 2 μg/mL of doxycycline were added into the culture medium after transfection for 12 h to induce the expression of the plasmids. After five-doses transfection for 11 days, the culture medium was changed to neuronal induction medium at Day 12. The neuronal induction medium contained Neurobasal Medium, N2, and B27 supplements, GlutaMAX, 1% of penicillin-streptomycin (all from Life technologies), and bFGF (100 ng/mL). To enhance the survival rate and improve the efficiency of direct conversion, the ROCK inhibitors Y27632 (2 μM), the P38 MARK inhibitors SB203580 (1 μM) and the BET family bromodomain inhibitors I-BET 151, (1 μM), sonic hedgehog (Shh, 500 ng/mL) and FGF8 (100ng/mL) were used. The medium (neuronal induction medium plus the small molecules) was refreshed every 3–4 days during the induction period.

### Quantitative Reverse Transcriptase Polymerase Chain Reaction

Quantitative reverse transcriptase polymerase chain reaction (qRT-PCR) was used to determine the messenger ribonucleic acid (mRNA) expression of cells after gene delivery. 1 mL of Trizol reagent (Invitrogen) was used to homogenize the cell lysate and the sample was left standing for 5 minutes at room temperature. Then, 200 μL chloroform was added into the mixture and centrifuged (13200 g, 5 minutes at 4 °C) to separate the phases. The transparent aqueous phase was transferred to a new tube and mixed with 500 μL isopropyl alcohol (Sigma-Aldrich, St. Louis, MO, USA). After the solution was vortex vigorously, RNA was collected by centrifugation (13500 g, 10 minutes at 4 °C). Then, the precipitate was washed with 1 mL of 75% ethanol. Next, the ethanol was removed by centrifugation, and the pellet was dissolved in 24 μL diethylpyrocarbonate-treated H_2_O (Life technologies, Carlsbad, CA, USA). The RNA was quantized by UltroSpec 3100 Pro (Amersham), and 1 μg of RNA was reversely transcribed with a SuperScript III reverse transcriptase kit (Invitrogen). qRT-PCR was performed in real time using an ABI 7900 Fast System and SYBR Green Master Mix. The real-time PCR cycling condition used was: 94 °C for 3 minutes, 35 thermal cycles (denaturing the DNA at 94 °C for 30 seconds, annealing with primers at 60 °C for 5 seconds, extending the length of the product at 72 °C for 1 minute), and a final extension of the product at 72 °C for 10 minutes. The reactions were carried out by specific primers and shown in Table S1. Moreover, glyceraldehyde3-phosphate dehydrogenase (Gapdh) was used to normalize the raw data of qPCR, and the error bar indicated the standard deviation of triplicate measurements.

### Immunofluorescence Staining of MFs-neuron

Immunofluorescence staining was used to identify the neuronal markers. After the direct conversion of MFs for 3, 7, 14, 19 days, the MFs-neuron were washed with PBS and fixed in 1% paraformaldehyde. Then, the MFs-neuron were permeabilized with 0.1% NP-40. Finally, the cells were incubated in the primary antibody Tuj1 (Abcam, ab76286), Dat (Abcam, ab111468), Tau (Abcam, ab76128), Th (Millipore, AB152), GABA (Abcam, ab17413), vGluT2 (Abcam, ab79157), NeuN (Abcam, ab177487) and synapsin I (Abcam, ab64581) against overnight. At Day 2^nd^, the cells were washed with PBS and treated with fluorescence-conjugated corresponding secondary antibodies (Jackson ImmunoResearch, 111-545-003, 315-545-003) and 4′,6-diamidino-2-phenylindole (DAPI). The MFs-neuron sample was observed under the fluorescence microscope.

### Measurement of dopamine release by enzyme-linked immunosorbent assay (ELISA)

The supernatant liquid of iM-Neurons was collected at Day 7^th^, 14^th^, and 22^th^. The following description was the experimental process.

#### Extraction and acrylation

Dopamine standards, controls and samples (750 μL/well) was added into coated plates, followed by 25μL TE buffer, and then shake for 60 minutes. After washed with wash buffer, 150 μL acrylation buffer and 25 μL acrylation reagent. After 20 minutes, it was washed with wash buffer again. Then 100 μL of hydrochloric acid was added into the plate and incubated for 10 minutes.

#### Enzymatic conversion

90 μL of standards, controls and samples were added into the microtitor plate, and 25 μμL of enzyme solution was added. Then, the microtitor plate was incubated at 37 °C for 2 hrs.

#### Dopamine ELISA

100 μL of standards, controls, and samples were added into dopamine microtitor plate, followed by dopamine antiserum and incubated at 2–8 °C for overnight (15–20 hrs). Then, it was washed with wash buffer and in which 100 μL of enzyme conjugate solution was added. Finally, 100 μL of substrate liquid was added for 20–30 minutes, and then 100μL stop solution was pipetted into each well. The absorbance of the solution in the wells was measured by a microplate reader at 450 nm within 10 minutes and the reference wavenumber was set between 620–650 nm.

### Electrophysiology

The electrophysiology experiments were done after the iM-Neurons for 22 days. MFs-neuron cells were placed in a submerged chamber with artificial cerebrospinal fluid (ACSF) containing the following (in mM): 125 NaCl, 25 NaHCO_3_, 1.25 NaH_2_PO_4_, 2.5 KCl, 25 glucose, 2 CaCl_2_, and 1 MgCl_2_. For the whole-cell patch-clamp recordings, recording electrodes (4–8 MΩ) were pulled from borosilicate glass tubing (outer diameter, 1.5 mm; inner diameter, 0.86 mm; Harvard Apparatus) filled with low Cl^−^ internal solution, containing the following (in mM): 136.8 K-gluconate, 7.2 KCl, 0.2 EGTA, 4 MgATP, 10 HEPES, 7 Na_2_-phosphocreatine, 0.5 Na_3_GTP (pH 7.3 with KOH). Individual cells were selected for the recordings based on a small round or ovoid cell body (diameters, 5–10 μm). The voltage-clamp recording was measured at a holding potential of −70 mV. The current-clamp was performed at −70 mV by using a holding current to adjust the membrane potential compatible. The tetrodotoxin (TTX, a voltage-gated Na+ -channel blocker, 1 *μ*M, Sigma) and 4-aminopyridine (4-AP, a voltage-gated A-type K^+^-channel blocker, 1 mM, Tocris) were used in some experiments.

## Electronic supplementary material

Supplementary Information
