# Supplementary material for: Generation of Functional Dopaminergic Neurons from Reprogramming Fibroblasts by Nonviral-based Mesoporous Silica Nanoparticles
Source: Sci Rep. 2018 Jan 8;8:11. doi: 10.1038/s41598-017-18324-8 (PMC5758610; doi:10.1038/s41598-017-18324-8)
Supplement: Supplementary file 1 — Supplementary Information [file 41598_2017_18324_MOESM1_ESM.doc]

**SUPPORTING INFORMATION**

**Generation of Functional Dopaminergic Neurons from Reprogramming Fibroblasts by Nonviral-based Mesoporous Silica Nanoparticles**

Jen-Hsuan Chang^a^, Ping-Hsing Tsai^b,c^, Kai-Yi Wang^d^, Yu-Ting Wei^d^, Shih-Hwa Chiou^b,c*^ and Chung-Yuan Mou^a*^

^a^Department of Chemistry, National Taiwan University, Taipei 106 Taiwan

^b^Department of Medical Research Taipei Veterans General Hospital, Taipei 112, Taiwan

^c^Institute of Pharmacology, National Yang-Ming University, Taipei 155 Taiwan

^d^Institute of Neuroscience, National Yang-Ming University, Taipei 155 Taiwan

**List of Table and Figures.**

**Table S1.** The zeta potential and DLS of (a): MSN-NH_2_, MSN-NH_2_ and MSN-NH_2_-biotin-avidin…………………………………………1

**Table S2.** The loading capacity of small molecular (Rh800, ISX-9) in nanoparticles……………………….1

**Table S3.** The primer sequence used in qRT-PCR analysis…………….1

**Figure S1.** Gel electrophoresis examination of DNA-MSN-avi complexes ………………………………………………2

**Figure S2.** Representative flow cytometry histogram of mouse fibroblasts (MFs) after incubation with FMSN-avi ……………………3

**Figure S3.** Confocal microscopy analysis of FMSN-avi uptake by MEFs…….………….……4

**Figure S4.** The cell viability assay………….…….……5

***Table S1. Primer sequence used in qRT-PCR analysis***

| **Gene** | **Primer sequence** **(5’** 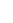 **3’)** |
| --- | --- |
| **Gapdh** | F: CTCATGACCACAGTCCATGC  R: TTCAGCTCTGGGATGACCTT |
| **Ascl1** | F: GCTGCAAACGCCGGCTCAAC  R: GCGGATGTACTCGACCGCCG |
| **Brn2** | F: CCATCGTACATGCCGAGCCGC  R: GCGCGGTGATCCACTGGTGAG |
| **Myt1l** | F: CGGGTGTGATGGAACCGGCC  R: GCCCTGTGCAGCCTGGAGTG |
| **Map2** | F: GAGATTGAGCTTCTGCCTACCAAC  R: GACATCATGGTCGCTGTAGTTCAG |
| **Shh** | F: CGGCAGATATGAAGGGAAGATC  R: TGCTCCCGTGTTTTCCTCAT |
| **Ngn2** | F: CCCATACAGCTGCACTTTATCG  R: AGGCGCATAACGATGCTTCT |
| **Vmat** | F: GCTACCCCACAGAAAGTGAAGATG  R: GATCAATGAAAGGCCCGCTT |
| **Dat** | F: GGGAGACCTGGAGCAAGAAAAT  R: GGCAATAACCATGAAGAGCAGG |

***Table S2.*** The zeta potential and DLS of (a): MSN-NH_2_, MSN-NH_2_ and MSN-NH_2_-biotin-avidin

|  | **Zeta potential**  **(mV, H_2_O, pH=7.4)** | **DLS size**  **(nm, H_2_O)** |
| --- | --- | --- |
| **MSN-NH_2_** | +30.4 (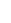1.13) | 184.6 (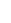2.57) |
| **MSN-NH_2_-biotin** | -28.7 (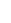0.58) | 207.6 (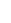3.35) |
| **MSN-NH_2_-biotin-avidin** | -16.8 (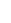0.60) | 383.5 (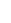3.18) |

***Table S3.*** The loading capacity of small molecular (Rh800, ISX-9) in nanoparticles

|  | **Small molecular loading capacity (**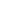**mol/g MSN)** |
| --- | --- |
| **ISX-9@MSN-NH_2_-biotin(+)-avidin** | 93.70 |

***
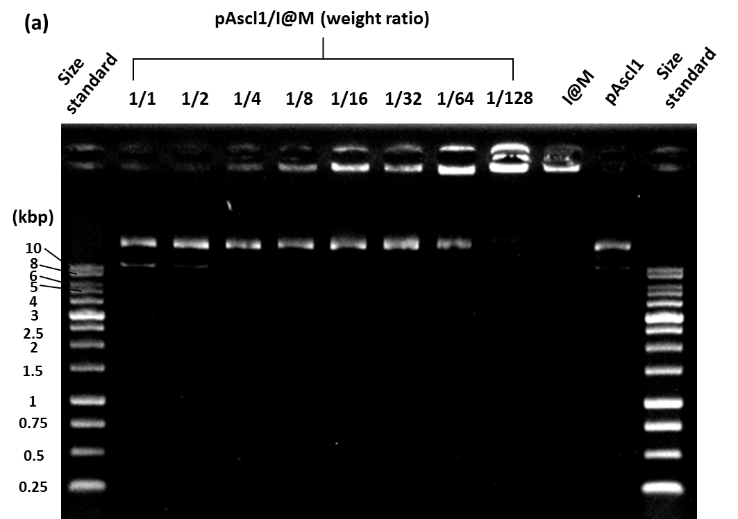
***

***
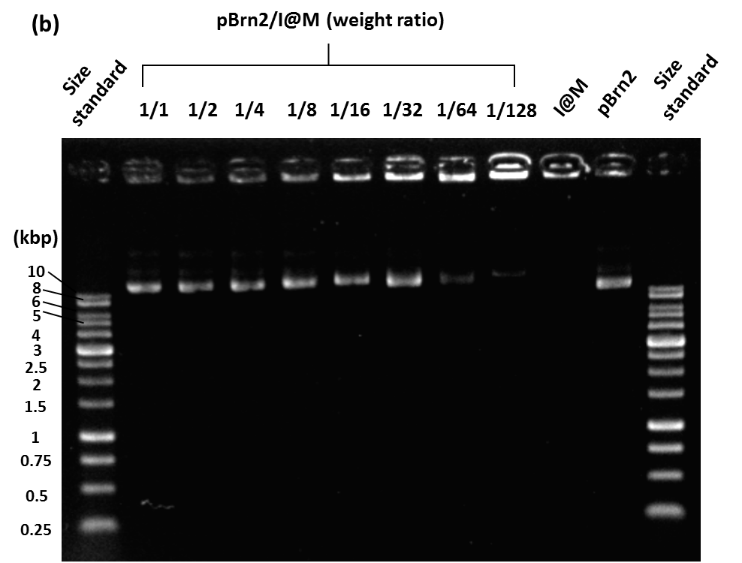
***

***
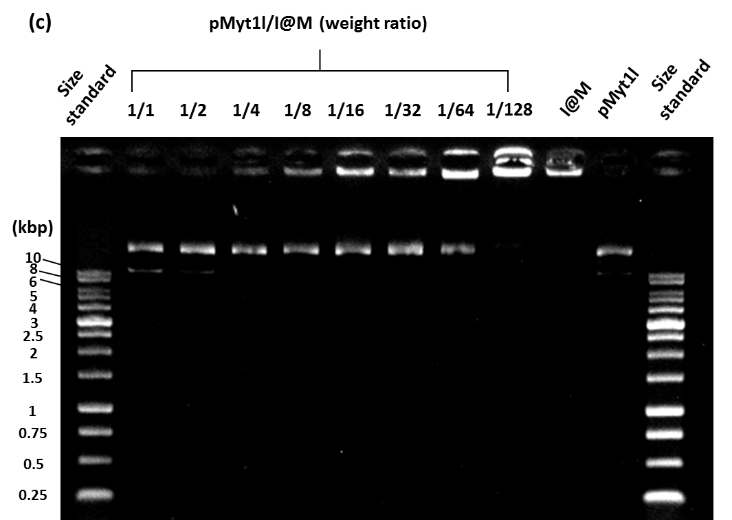
***

***Figure S1.*** Gel electrophoresis examination of DNA-MSN-avi complexes: (a) pAscl1-I@M, (b) pBrn2-I@M and (c) pMyt1l-I@M in 1% agarose gel. The DNA ladder, DNA and I@M were references.

***
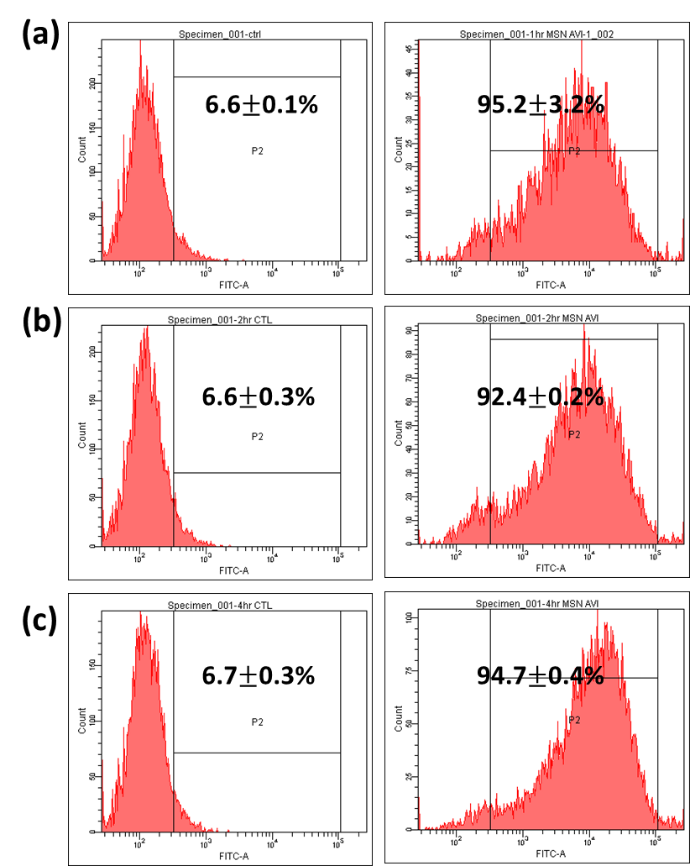
***

***Figure S2.*** Representative flow cytometry histogram of mouse fibroblasts (MFs) after incubation with FMSN-avi (256 μg/mL) for (a) 1 hr, (b) 2 hr and (c) 4 hr.


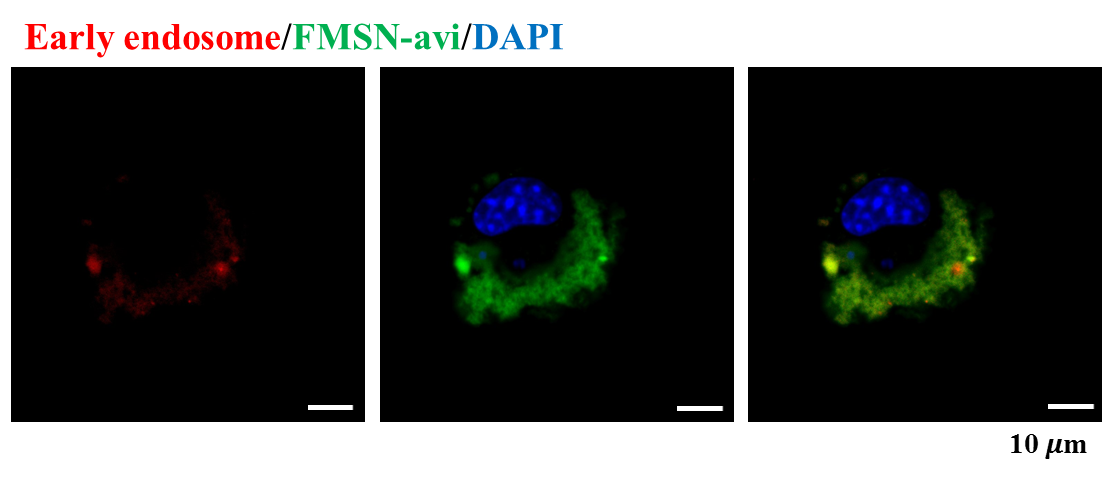


***Figure S3.*** Confocal microscopy analysis of FMSN-avi uptake by MFs: Left: FM4-64 stained the endosome (red); Middle: FMSN-avi (green) and DAPI (4', 6-diamidino-2-phenylindole) stained the nucleus (blue); Right: FMSN-avi (green), endosome (red) and nucleus (blue).


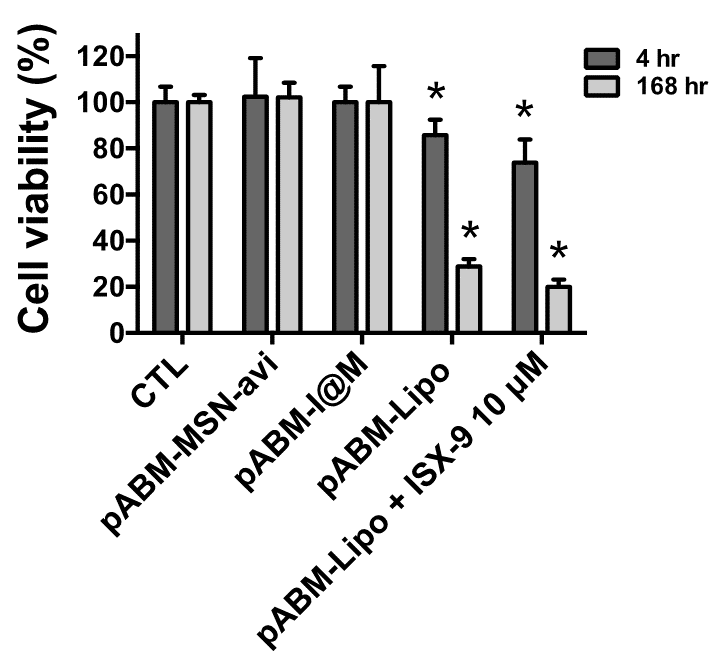


***Figure S4.*** Cell viability assay. Delivery of pABM-I@M, pABM-MSN-avi, pABM-Lipo+10μM ISX-9 and pABM-Lipo into MFs to evaluate the cell cytotoxicity (4 hr) and cell proliferation (96 hr).The cell viability was examined by calculating the cell number after the cells were trypsinized. (amount of pABM: 2 μg/well; amount of Lipofectamine 2000: 6 μL/well; amount of I@M, MSN-avi: 256 μg/well)
